# Severe duodenal entrapment secondary to omental adhesion in a Labrador retriever

**DOI:** 10.1111/jsap.70056

**Published:** 2025-11-28

**Authors:** L. F. Doeven, S. Barker, L. Cole, F. O. Buishand

**Affiliations:** ^1^ Department of Clinical Science and Services Royal Veterinary College Hatfield UK

A nine‐year‐old male neutered Labrador presented with an acute history of regurgitation, vomiting and lethargy. The dog had a previous diagnosis of chronic inflammatory enteropathy for which he was receiving 0.8 mg/kg prednisolone PO once a day, alongside elevation of liver enzymes of unknown relevance/cause. At presentation, haematology was unremarkable and biochemistry revealed ongoing elevation of liver enzymes (ALT 239 U/L, ALP 302 U/L). Abdominal radiographs were taken, showing a slightly gas‐distended stomach in the left cranial abdomen (Fig 1A). The right lateral projection demonstrated continuation between the partially gas‐distended pylorus into a markedly distended loop of duodenum and a second mid‐abdominal loop (Fig 1A). The remaining small intestines were considered within normal limits. These changes were suggestive of a proximal small intestinal mechanical obstruction. The primary differentials were mesenteric rent, torsion, stricture or obstructive intestinal disease. The dog had no prior history of abdominal surgery. An exploratory coeliotomy revealed marked duodenal dilation, with the omentum covering the duodenum upon entry. The liver was generally enlarged with a roughened surface. The duodenum was entrapped within an enclosure created by an omental adhesion to the caudate process of the caudate liver lobe (Fig 1C), with the entrapped duodenum accounting for both distended loops visible on the radiographs. No torsion, nor discolouration of the entrapped intestine was present. The adhesion was broken down with electrocautery and the duodenum released. Some dilation was relieved via passing of a orogastric tube. Complete decompression was not possible, which was due to luminal contents blocking the needle hub, or the marked distension making manipulation of the gaseous contents challenging. The dog recovered uneventfully. Despite the novelty of this case, it reiterates the diagnostic utility of radiographs for diagnosing gastrointestinal obstruction when considering an exploratory celiotomy.

**FIG 1:**
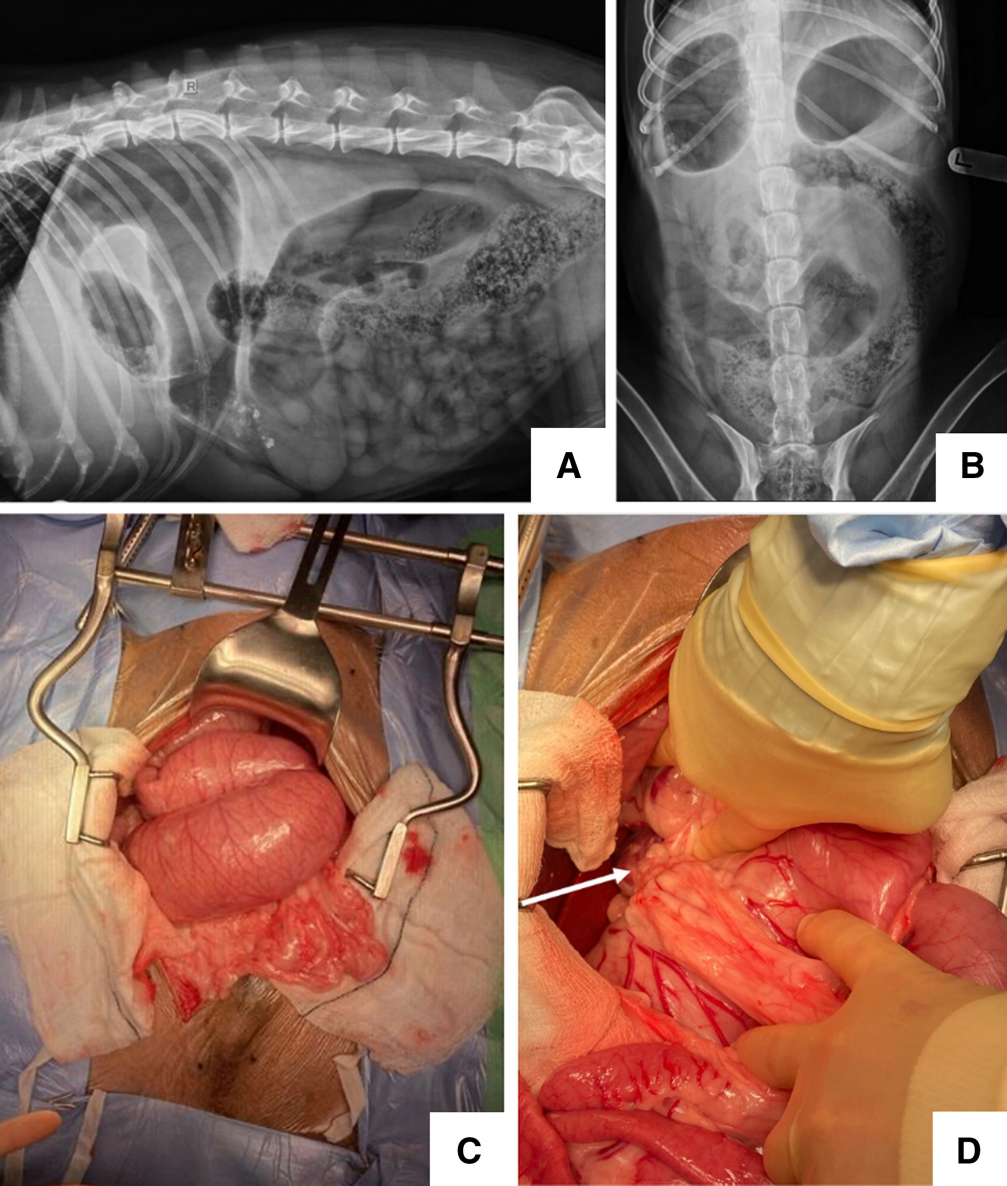
Right lateral (A) and ventrodorsal (B) abdominal radiographs. Intraoperative photos: marked duodenal distension (C) and omental adhesion (white arrow) (D).

## Author contributions

L. F. Doeven drafted the manuscript. All authors contributed equally to the management of the case and revising of the manuscript.

